# Development and Evaluation of a Leukemia Diagnosis System Using Deep Learning in Real Clinical Scenarios

**DOI:** 10.3389/fped.2021.693676

**Published:** 2021-06-24

**Authors:** Min Zhou, Kefei Wu, Lisha Yu, Mengdi Xu, Junjun Yang, Qing Shen, Bo Liu, Lei Shi, Shuang Wu, Bin Dong, Hansong Wang, Jiajun Yuan, Shuhong Shen, Liebin Zhao

**Affiliations:** ^1^Pediatric AI Clinical Application and Research Center, Shanghai Children's Medical Center, Shanghai, China; ^2^Department of Hematology, Shanghai Children's Medical Center, Shanghai, China; ^3^Shanghai Key Laboratory of Artificial Intelligence for Medical Image and Knowledge Graph, Shanghai, China; ^4^YITU AI Research Institute for Healthcare, Zhejiang, China; ^5^Department of Laboratory Medicine, The Second Affiliated Hospital and Yuying Children's Hospital of Wenzhou Medical University, Zhejiang, China; ^6^Children Health Advocacy Institute, China Hospital Development Institute of Shanghai Jiaotong University, Shanghai, China; ^7^Division of Medical Administration, Shanghai Children's Medical Center, Shanghai, China

**Keywords:** deep learning, leukemia, morphological diagnosis, artificial intelligence, computer-aided diagnose

## Abstract

Leukemia is the most common malignancy affecting children. The morphologic analysis of bone marrow smears is an important initial step for diagnosis. Recent publications demonstrated that artificial intelligence is able to classify blood cells but a long way from clinical use. A total of 1,732 bone marrow images were used for the training of a convolutional neural network (CNN). New techniques of deep learning were integrated and an end-to-end leukemia diagnosis system was developed by using raw images without pre-processing. The system creatively imitated the workflow of a hematologist by detecting and excluding uncountable and crushed cells, then classifying and counting the remain cells to make a diagnosis. The performance of the CNN in classifying WBCs achieved an accuracy of 82.93%, precision of 86.07% and F1 score of 82.02%. And the performance in diagnosing acute lymphoid leukemia achieved an accuracy of 89%, sensitivity of 86% and specificity of 95%. The system also performs well at detecting the bone marrow metastasis of lymphoma and neuroblastoma, achieving an average accuracy of 82.93%. This is the first study which included a wider variety of cell types in leukemia diagnosis, and achieved a relatively high performance in real clinical scenarios.

## Introduction

Leukemia, which results from the maturation arrest and differentiation block of nucleated cells and can cripple the production of normal blood cells, may present at all ages, from newborns to very old people, and it is the most common malignancy affecting children, representing up to 30% of all pediatric cancers (1). Moreover, these immature cells can spread into the blood and invade other organs, leading to the dysfunction of multiple organs and eventual death. Because of the rapid proliferation and fast dissemination of leukemia cells, early and accurate diagnosis is urgently needed.

Despite the wide use of immunological, cytogenetic, and molecular tests, the morphologic analysis of bone marrow smears is still an important initial step for leukemia diagnosis (2, 3), as it is an economical and relatively convenient method. Morphologists analyses the characteristics of blood cells, such as shape, size, and granularity to define the cell types by using a light microscope, and then, they make diagnoses according to the guidelines. However, classical morphological diagnosis is tedious and labor-intensive work, which involves time and highly trained professionals, and the diagnosis results may be subjective. In contrast, a computer-aided diagnosis (CAD) system can help save time and overcome the shortcomings of manual work including exhaustion, subjectivity and so on.

The differential count of white blood cells (WBCs) is the base of the morphological diagnosis of leukemia. Computerized analysis based on deep learning has shown potential promise as a diagnostic strategy for the differential count of WBCs. Choi et al. (4) and Qin et al. (5) demonstrated the potential of deep learning for classifying WBCs in different stages of maturation, which making deep learning-based leukemia diagnosis possible, however, these studies had limitations due to few cell types and low accuracy, respectively, and the classification was usually performed using pre-processed images, rather than raw clinical images. The differential count of WBCs for bone marrow analysis is an important application of deep learning, but it requires improvement.

In this study, bone marrow images of children with leukemia were retrospectively collected from the Shanghai Children's Medical Center, and the WBCs were annotated. We used the results to establish a leukemia cell database, which is named AI-cell platform. The cell images in the database were used for training and testing to develop a leukemia diagnosis system using deep leaning to discriminate up to 19 WBC types in different stages of maturation in real clinical scenarios rather than using pre-processed images or online public data-sets. The differentiated leukocyte types were able to satisfy the requirement for the diagnosis of common childhood leukemia. Our system imitated the process of bone marrow smear analysis by hematologists. Moreover, we further evaluated the potential of the artificial intelligence system for diagnosing acute lymphoblastic leukemia (ALL) in clinical work.

## Materials and Methods

### Image Datasets

To enable the development of diagnostic machine learning algorithms, we established a database, the AI-cell platform, which consists of 1,732 images obtained from the bone marrow smears of 89 children with leukemia from 2009 to 2019 at the Shanghai Children's Medical Center (SCMC). The bone marrow smears involved were stained according to the Wright-Giemsa protocol. The images of the prepared slides were acquired with a light microscope at x1000 magnification. For each smear, 15 non-overlapping acquisition locations on average were randomly selected. The need for informed consent was waived by the institutional review board of the SCMC. All the images were deidentified before being made available. The images used in this study were produced with a camera (MooGee; 505 C GS).

### Reference Standard

**Figure 1:**
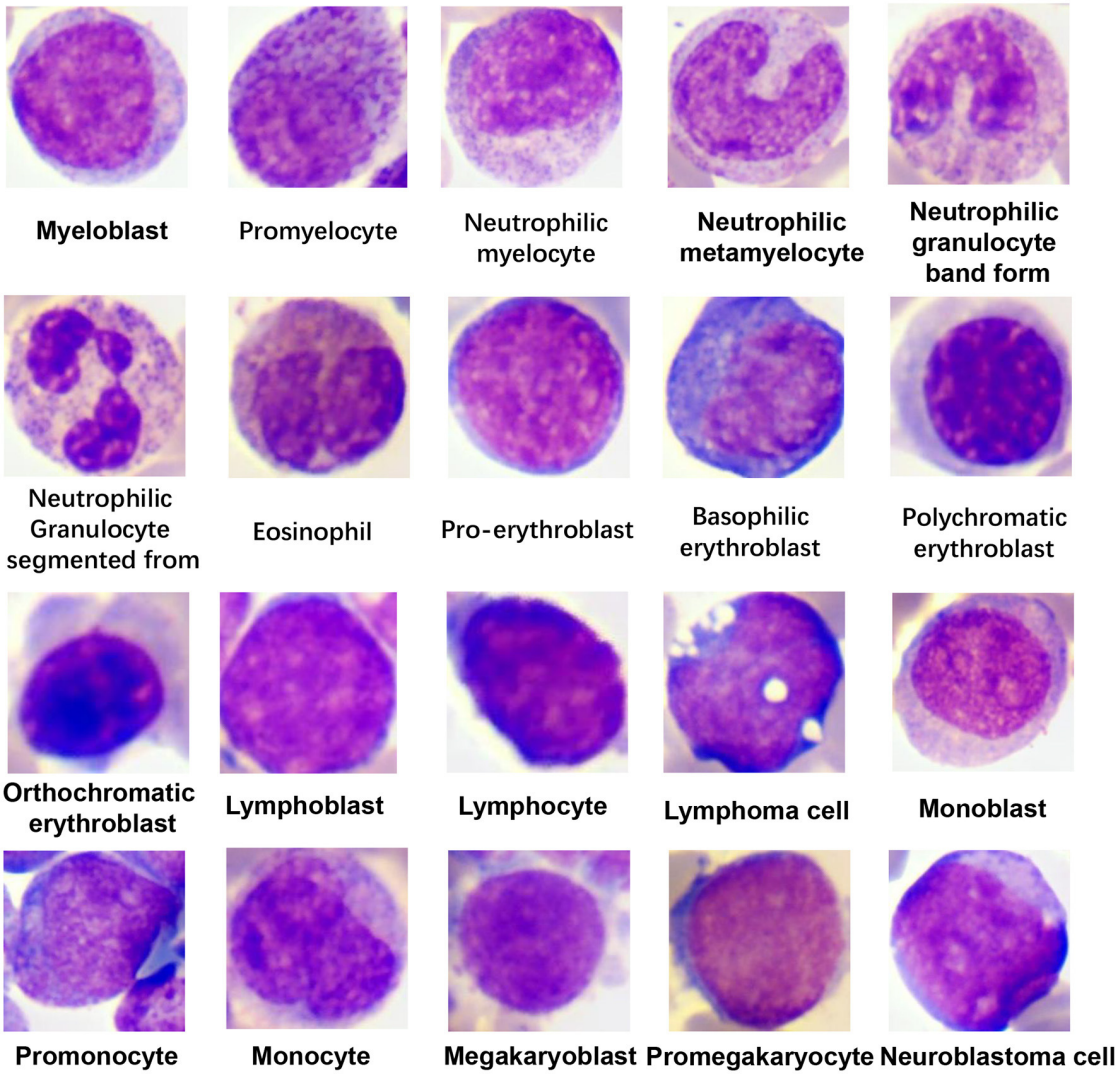
Representative images of the classified cell types.

**Figure 2:**
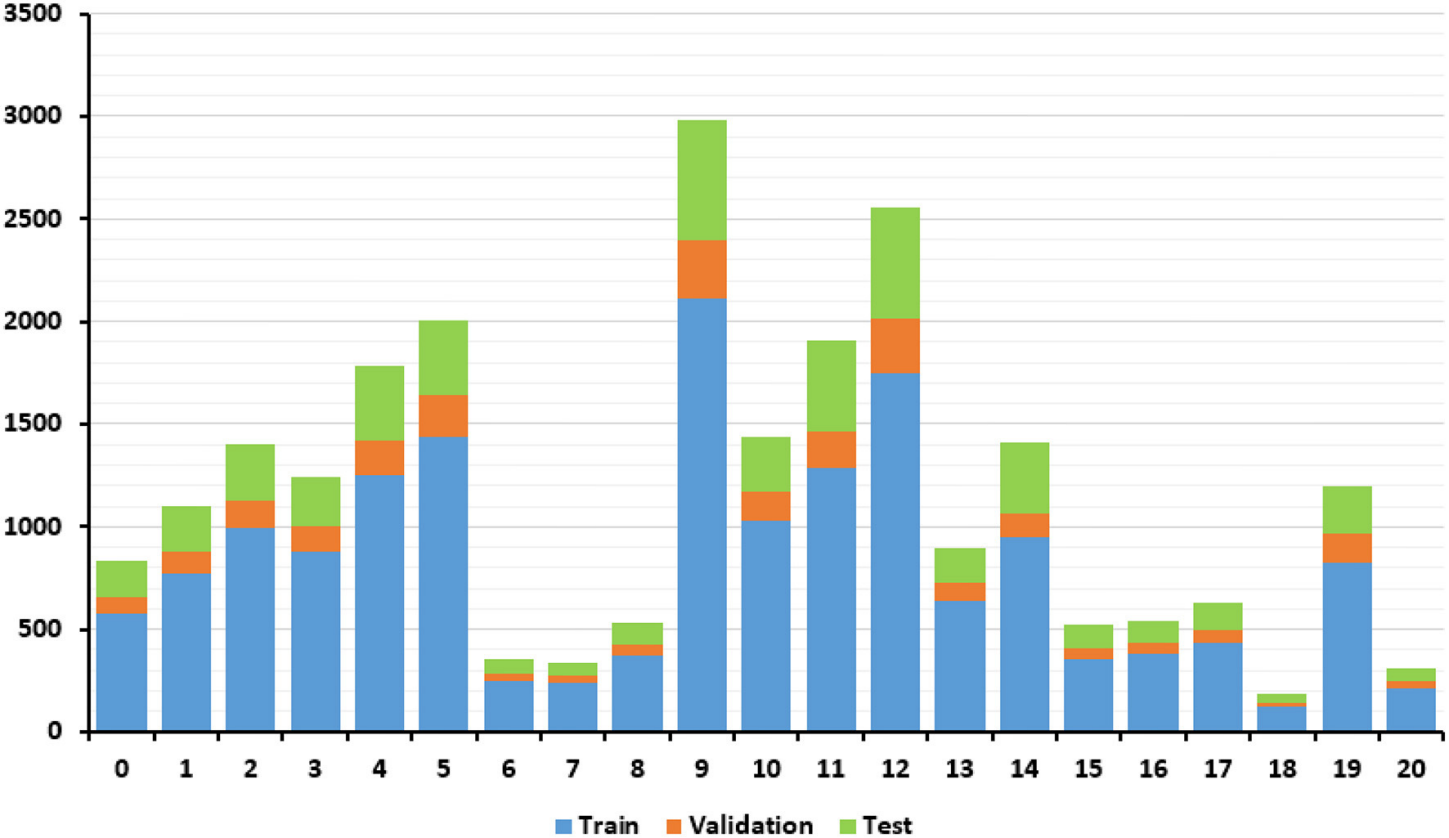
Dataset split and distribution of categories.

The corresponding diagnoses of each bone marrow smear were confirmed via flow cytometry. The exact types of WBCs in each image were annotated by 2 hematologists who have worked for more than 10 years, and they checked each other's annotations to ensure accuracy. The dataset was composed of 19 WBC classes in different maturation stages and neuroblastoma cells (Figure 1). In fact, there are up to 40 types of WBCs in a bone marrow image, and some types of WBCs account for very small proportions. We could not collect enough images of these infrequent WBCs to train the model. Therefore, we combine these cells into a group (class 20) during the training of the classification model. The number of cells for each class and their distribution are shown in Figure 2 and Table 1. The distribution is imbalanced among classes. Since the natural distribution of WBCs is imbalanced, this problem was unavoidable.

**Table 1:** The cell types and total numbers of cells of each class in the dataset.

**Class**		**Category**	**Total number**
Granulocyte	0	Myeloblast	830
	1	Promyelocyte	1,101
	2	Neutrophilic myelocyte	1,398
	3	Neutrophilic metamyelocyte	1,244
	4	Neutrophilic granulocyte band form	1,784
	5	Neutrophilic granulocyte segmented from	2,004
	6	Eosinophil	352
Erythroid	7	Pro-erythroblast	337
	8	Basophilic erythroblast	530
	9	Polychromatic erythroblast	2,983
	10	Orthochromatic erythroblast	1,439
Lymphocyte	11	Lymphoblast	1,905
	12	Lymphocyte	2,558
	13	Lymphoma cell	900
Monocyte	14	Monoblast	1,410
	15	Promonocyte	520
	16	Monocyte	544
Megakaryocyte	17	Megakaryoblast	630
	18	Promegakaryocyte	185
Others	19	Neuroblastoma cell	1,201
	20	Uncountable cells	310

*The uncountable cells included reticular cells, mast cells, naked nuclei and so on, which are usually not involved in the diagnosis of leukemia*.

### Training of CNN

**Figure 3:**
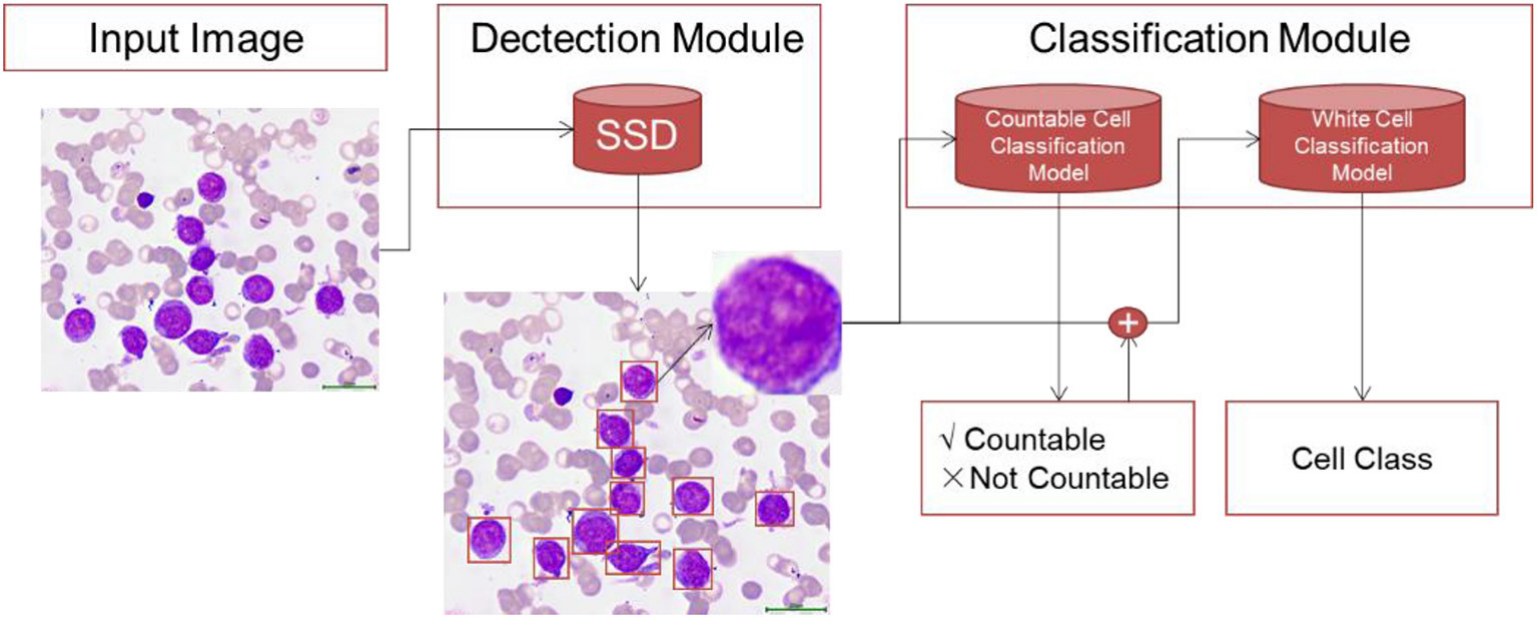
The overall modeling framework containing two modules: detection module and classification module. In the detection module, we train detection model using RetinaNet method to detect all the WBCs in bone marrow images. The classification module contains two stage. In the first stage, we develop a countable cell classification model to discriminate crush white blood cell which would not be counted by hematologists. Then in the second stage, the detected countable white blood cells are submitted to classification model for WBC classification.

The WBC differential count system contained two modules: the detection model and the classification model. The raw bone marrow smear images were first processed by the detection module, through which all the WBCs were detected from red blood cells, blood platelets, staining impurities and so on. Then, the detected cells were used as input for the classification module. The classification module contained two stages. In the first stage, we discriminated the uncountable cells including crush cells, degenerated cells and so on, which are not used in the diagnosis of leukemia. In the second stage, the countable WBCs were submitted for multi-class differentiation (Figure 3).

We used RetinaNet (6) with VGG (7) and the Feature Pyramid Network (8) for cell detection. RetinaNet is a one-stage detector which surpasses the performance of all existed state-of-the-art two-stage detectors. Supplementary Figure 1 shows the framework of RetinaNet. The network composed of a backbone network and two subnetworks. The backbone network is used for feature computing, and the subnets are used for bounding box regression and classification. FPN was adopted as the backbone network. It includes a top-down pathway (VGG) and lateral connections. This structure could generate multi-scale feature pyramid from the input image. There are two subnets attached at each FPN level: classification subnet to predict the probability of object pretension for each anchor and each object class, and box regression subnet to estimate the offset from each anchor box to a nearby ground-truth object. Both subnets are small fully convolutional networks (FCN). The object classes in this work are cell and background.

In this study, we adopted the ResNet (9) method to propose WBC classification model. ResNet is a widely used deep learning framework for image classfication. The layers in ResNet were reformulated to learn the residual function. This residule structure could train a deeper network efficiently. Similar to ResNet, ResNeXt (10) was constructed by repeating a building block that aggregates a set of transformations with the same topology. The network introduces a new dimension “cardinality,” which indicates the size of the set of transformations. ResNeXt could generate deeper and wider deep learning models. Supplementary Figure 2 shows the a ResNet block and a ResNeXt block. It could be seen that ResNet learns the residule of the net, and ResNeXt block is wider than ResNet block. Supplementary Figure 3 shows the structure of ResNet 50 and ResNeXt50 (32x4d). ResNext101 32^*^8d is adpoted in stage1 classification, and the ensemble model of ResNext101 32^*^8d, ResNext50 32^*^4d and ResNet50 is used for stage2 classification.

The entire dataset was split image-wise into a training set (70%), validation set (10%), and test set (20%). The training set was used to train the detection and classification models. The validation set was used to choose the best parameter for each model. The test set was applied to evaluate the trained model. All stages used the same data split.

The weights of each layer were initialized using semi-weakly supervised ImageNet mode (11). For both stage 1 and stage 2, Adam optimizer (12) with cosine annealing learning rate scheduler (13) is applied during training process. For stage 1 classification, the initial learning rate = 1e-5, batch size = 32, and T max of cosine annealing learning rate scheduler = 30. For stage 2 classification, the initial learning rate = 1e-5, batch size = 32, and T max of cosine annealing learning rate scheduler = 50. The hyperparameters were determined using validation set via grid search.

### Statistical Analysis

The trained network was tested on the test dataset and the classification performance was assessed quantitatively through the following metrics: mean accuracy, precision, recall, and F1 score,

$$\begin{array}{lll} Accuracy & = & \frac{T_{P}+T_{N}}{T_{P}+F_{P}+T_{N}+F_{N}}\\ Precision & = & \frac{T_{P}}{T_{P}+F_{P}}\\ Recall & = & \frac{T_{P}}{T_{P}+F_{N}}\\ F1-score & = & 2\cdot \frac{Precision\cdot Recall}{Precision+Recall} \end{array}$$

where TP is the number of true positive classifications, TN is the number of true negatives, FP is the number of false positive classifications, and FN is the number of false negatives. A confusion matrix of the classes was also created to analyse the class-wise performance.

## Results

In this study, we proposed a deep learning system to imitate the WBC differential count process conducted by hematologists in real clinical scenarios. In the collected raw images of bone marrow smears, there are many staining impurities, cell debris and blood platelets, which make segmentation of bone marrow cells difficult. We proposed a WBC differential system containing a three-stage deep learning model to detect and classify the WBCs in bone marrow images. First, we trained an SSD model to detect all WBCs in the bone marrow images. Then, we developed a ResNet model to discriminate crushed WBCs, as these cells would not be included in the differential counting during the working process of hematologists. Finally, we developed a deep learning model to classify multiple types of WBCs to realize the artificial intelligence diagnosis of leukemia. By counting the classified cells, diagnoses can be made according the FAB classification.

### Detection of Countable WBCs

In the first stage of our WBC counting system, we used the SSD method to form a deep learning model to detect all WBCs in the bone marrow images. As a single-shot multi-box detector for multiple categories, the SSD method can be decomposed into a truncated base network and several auxiliary convolutional layers used as feature maps and predictors. SSD has achieved excellent performance according to the trade-off between the detection accuracy and speed. Our proposed SSD model achieved good performance, with AP = 0.9348 for detecting WBCs.

**Figure 4:**
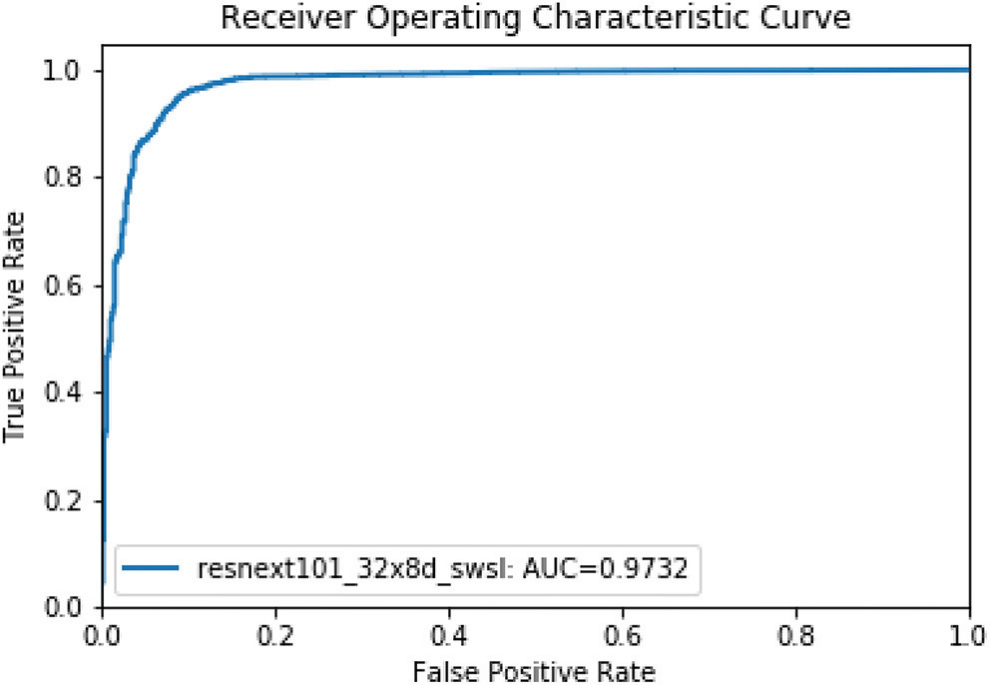
The ROC curve of the second model stage for discriminating countable and uncountable WBCs.

Then, in the second stage, we proposed a deep learning model using the ResNet method to discriminate uncountable white blood cells (including reticular cells, mast cells, naked nuclei and so on) and the countable cells that are ultimately counted for bone marrow analysis. The ResNet model also achieved good performance. The accuracy, precision, and recall of the model were 0.9656, 0.9797, and 0.9837, respectively. We illustrate the ROC curves (AUC = 0.9732) of the deep learning model in Figure 4.

### Multiclass WBC Classification

For multi-class WBC differentiation, we developed three deep learning models (ResNext101_32^*^8d swsl, ResNext50_32^*^4dswsl and ResNet50) using the ResNet method with various architectures and parameters. Next, to further improve the performance of the WBC differential counting system, we combined the three ResNet models to propose an ensemble model. The ensemble model combines the decisions from the multiple models to improve the overall performances. The ensemble method could help to minimize the factors that cause errors, such as noise, bias and variance, and the predictions for the single model were averaged to obtain the final prediction.

**Figure 5:**
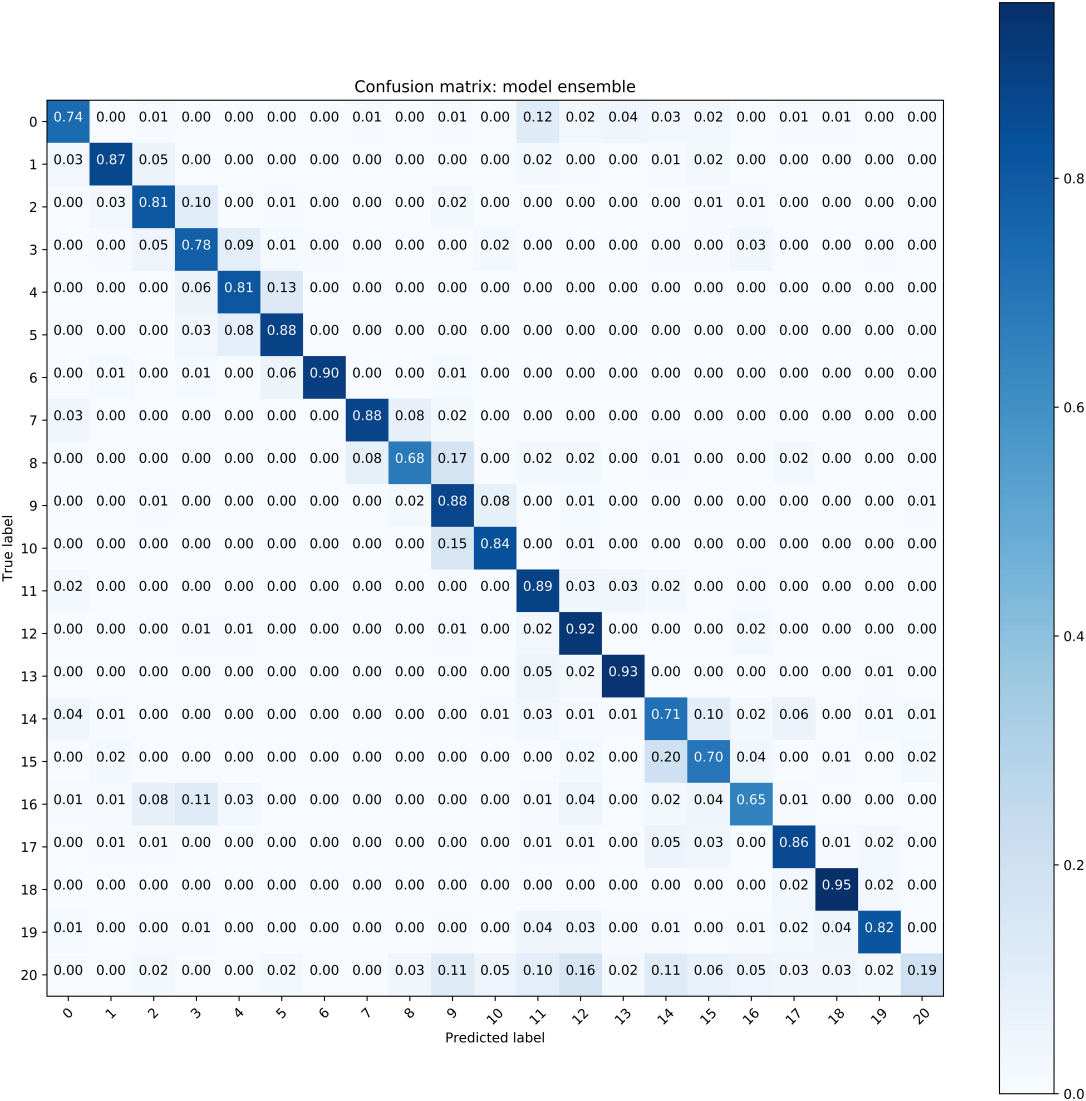
The confusion matrix of the ensemble model for WBC classification (Refer to Table 1 for cell types of each class).

The performance of all these models is illustrated in Table 2, where we used the average accuracy, AP, F1 score and AUC as the main metrics. The ensemble model exhibited better performance than the three Resnet models. For the classification of the 19 types of WBCs, the ensemble model achieved good performance, with an average accuracy of 0.8293, AP of 0.8567, F1 score of 0.8293 and AUC of 0.9870. A confusion matrix of the classification results from the ensemble model on the test dataset was generated to evaluate the class-wise performance (Figure 5). Among these types of WBCs, our model achieved a sensitivity of over 90% for eosinophils, lymphocytes, lymphoma cells, and promegakaryocytes. The sensitivity of neuroblastoma cells, promyelocytes, neutrophilic myelocytes, neutrophilic granulocyte band forms, neutrophilic granulocyte segmented forms, pro-erythroblasts, polychromatic erythroblasts, orthochromatic erythroblasts, lymphoblasts, and megakaryoblasts was between 80 and 90%. In total, 14 of the 20 types of cells achieved a sensitivity of over 80%. The majority of misclassifications occurred with the basophilic erythroblasts and the monocytes, achieving 68 and 65% sensitivity, respectively. The performance differences between the different types of WBCs mainly resulted from the various sizes of the datasets since the lack of large datasets of carefully annotated cells has been the limitation to improving medical image recognition systems (14).

**Table 2:** The performance of the deep learning model for the classification of types of WBCs.

**Model**	**Accuracy**	**AP**	**F1 score**	**AUC**
ResNext101_32^*^8d swsl	0.8149	0.8441	0.8149	0.9856
ResNext50_32^*^4dswsl	0.7982	0.8327	0.7982	0.9844
Resnet50	0.8073	0.8023	0.8074	0.9813
Ensemble model	0.8293	0.8567	0.8293	0.9870

**Figure 6:**
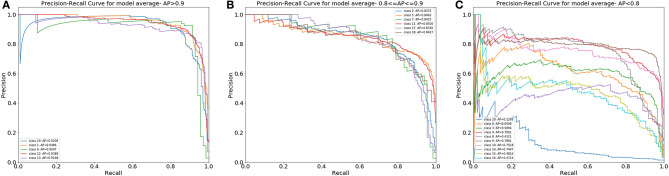
The PR curves of the differential count of 20 types of cells. **(A)** Cell classes with an AP over 0.9. **(B)** Cell classes with an AP from 0.8 to 0.9. **(C)** Cell classes with an AP under 0.8.

Moreover, we illustrated the performance of the ensemble model in classifying single types of WBCs by using the precision-recall curve (Figure 6). Our model achieved APs of over 0.9 for classes 1, 6, 12, 13, and 19. The APs of classes 2, 5, 7, 11, 17, and 18 ranged from 0.8 to 0.9. In general, the proposed model could accurately differentiate promyelocytes, lymphoblasts, and promegakaryocytes, which covered all related WBCs for the diagnosis of ALL, AML (M3 and M7) and the bone marrow metastasis of lymphoma and neuroblastoma. For class 20, this group contains many types of cells, and there are not enough images of them for training; therefore, our model achieved poor performance for this group.

### Diagnosis of Acute Lymphoid Leukemia

To show the potential of the method in clinical applications, we tested the CNN in newly diagnosed ALL patients in 2020 in the SCMC. Different from previous studies in which single lymphoid cells were defined as benign or malignant, our deep learning model “read” about five non-overlapping scopes of bone marrow smears of each patient and made the diagnosis of ALL if the percentage of lymphoblasts was over 20% according to the FAB classification (15). We retrospectively collected data from 49 patients (24 ALL and 25 AML) to evaluate the potential of our model to diagnose ALL. The proposed model achieved significant performance, with an accuracy of 0.89, sensitivity of 0.86 and specificity of 0.95.

## Discussion

In this study, we retrospectively collected 1,732 bone marrow images containing 27,184 cells (including 24,165 cells and 2,983 cell debris) from 89 children with leukemia from the Shanghai Children's Medical Center. We randomly separated 70% of the cells in the training set, and the remaining cells were used to form the validation set and test set. This research aimed to develop an end-to-end leukemia diagnosis system using deep learning to discriminate up to 19 types of WBCs, which could cover enough types of WBCs for the diagnosis of childhood leukemia. The system imitated the workflow of a hematologist. First, the system detects all the white blood cells without classification, achieving good performance, with AP = 0.9348. Then, we proposed a dichotomous model to discriminate crushed white blood cells and countable cells, which were finally counted for bone marrow analysis. The accuracy, precision and recall were 96.76, 97.97, and 98.37%, respectively. Finally, the countable cells were submitted to a classification model, which achieved an accuracy of 82.93%, precision of 86.07% and F1 score of 82.02%. In addition, we tested the algorithm's performance in diagnosing ALL, achieving an accuracy of 0.89, sensitivity of 0.86 and specificity of 0.95. We also tested the system at detecting the bone marrow metastasis of lymphoma and neuroblastoma, achieving an accuracy of 0.8293, AP of 0.8567, F1 score of 0.8293 and AUC of 0.9870. Importantly, the proposed model achieved an accuracy over 80% for all WBCs related to the diagnosis of leukemia.

The differential count of WBCs is the first step in the automatic recognition of different types of leukemia, and there have been several studies about the differential count of WBCs. However, there is some room for improvement. Yusuf Yargi Baydilli Jin's capsule networks effectively learned training data and achieved a high accuracy on the test data (96.86%), but WBCs were only classified under five categories (16). Choi et al. (4) developed a WBC differential count system using a dual-stage CNN and achieved high performance. However, only 10 types of cells were involved in that study, which is far from enough to diagnose leukemia. Qin's (5) research tried to classify up to 32 types of WBCs, but the average accuracy was low because of the limited data. To the best of our knowledge, this is the first study that aimed to establish a leukemia diagnosis system with abundant cell types and achieved relatively high accuracy (Table 3). Our study established a larger database that consists of more WBCs related to leukemia diagnosis than other studies (27).

**Table 3:** Comparison of the classification accuracy of different researches in WBC detection.

**Literature**	**Dataset**	**Number of candidate cells**	**No. of WBC types**	**Diseases involved**	**Technique used**	**Accuracy achieved**
MoradiAmin et al. (17)	Real world	958	2	ALL	Feature extraction, c-means	>90% (disease level)
Bigorra et al. (18)	Real world	916	3	ALL, AML	Feature extraction	>74% (cell level)
Choi et al. (4)	Real world	2,174	10	ALL, AML	CNN	97.06% (cell level)
Shafique et al. (19)	ALL-IDB	108+260	2	ALL	CNN	>95% (disease level)
Moshavash et al. (20)	ALL-IDB	108+260	2	ALL	Segmentation, feature extraction, SVM	89.81% (disease level)
Qin et al. (5)	Real world	92,480	40	ALL, AML	CNN	76.84% (cell level)
Rehman et al. (21)	Real world	/	2	ALL	Segmentation, CNN	97.78% (disease level)
Boudu et al. (22)	Real world	7,468	6	ALL, AML	Feature extraction	85.8% (cell level)
Shahin et al. (23)	ALL-IDB	108+260	2	ALL	Feature extraction, CNN	96.1% (cell level)
Anwar et al. (24)	ALL-IDB	108+260	2	ALL	CNN	99.5% (disease level)
Gehlot et al. (25)	TCIA	15,114	2	ALL	CNN	94.8% (F1 score, cell level)
Zhang et al. (26)	BCCD	5,000	6	/	CNN, HOG, SVM	>95% (cell level)

Generally, an automatic WBC recognition system usually involves extracting effective features (28). These hand-engineered features, such as geometrical, color or texture features, have proven to be subjective and less effective than features learned by CNNs (29). Therefore, an important high-level decision in our approach was to learn features using deep CNNs rather than classical hand-engineered features. Moreover, although real-world images were used in some previous studies (4, 5), they were pre-processed to remove the background impurities and annotate single cells, which is very different from real clinical scenarios. These studies failed for prove their performance in real clinical scenarios. Therefore, different from previous studies, our research focused on clinical applications, and the images that we have assessed were collected from a local hospital rather than an online public database and were not specially processed. In addition, we have proven that a CNN trained by a complex, raw set could work very well in real clinical scenarios. Our study achieves outstanding performance at detecting and classifying WBCs in complex real clinical scenarios. More specifically, we were interested in replicating a more complete part of a hematologists' workflow, including three steps, namely, the detection of WBCs, the exclusion of crushed cells and the final classification.

Previous studies about leukemia diagnosis mainly focused on ALL. The average accuracy for detecting and classifying ALL cells is over 90.00% (27). There are also other studies that focus on the diagnosis of types of leukemia. Laura Bigorra and her colleagues (18) published their first research in 2016, in which their overall classification accuracy and the true-positive rates for reactive lymphoid cells, myeloid blast cells and lymphoid blast cells were 80, 85, 82, and 74%, respectively. In 2019, this group successfully diagnosed six classes of blood smears (ALL-B, M3 type AML, other types of AML, Infections, Control-lymphocyte and Control-Monocyte), achieving an accuracy of 94% (22). In that article, the authors classified M3 type AML as different from AML; however, they failed to classify other types of AML. Our research aimed to achieve the comprehensive diagnosis of multiple types of leukemia, so the types of WBCs cells collected were involved in as many types of leukemia as possible. We tested the deep learning model in clinical work for the diagnosis of ALL, which achieved an accuracy of 0.89, sensitivity of 0.86 and specificity of 0.95. A distinctive feature of our study is that the diagnosis was made according to the FAB classification, in which the ALL diagnosis could be made when the percentage of lymphoblasts among karyocytes was over 20%. Different from previous studies which have not developed a counting function and just judged single cells as benign or malignant or which have used online database with pre-processed images as training set, our system focused on clinical application in real world.

Because of the lack of sufficient cases of AML, we were not able to realize the automatic diagnosis of myeloid leukemia. As shown in the PR curve of the classification results.

The classification model achieved good performance for the differentiation of promyelocytes, lymphoblasts, and promegakaryocytes, implying that M3 and M7 types can be diagnosed with high accuracy.

To assess the clinical application of the CNN, we tested its performance on the detection of bone marrow metastasis for two types of solid tumors, lymphoma and neuroblastoma. Bone marrow is the most common site of infiltration in children with neuroblastoma presenting with metastatic disease at the time of diagnosis, is a frequent site of the disease's recurrence and is predictive of poor outcomes (30). In addition, for lymphoma, bone marrow evaluation plays a critical role in staging and predicting the prognoses in patients with this di and bone marrow can be the initial detection site of lymphoma in patients with unexplained symptoms or cytopenia (31). Therefore, it is of great clinical significance to judge the bone marrow metastasis of these two types of tumors. The results showed that our CNN could work very well in the detection of bone marrow involvement in patients with neuroblastoma and lymphoma, and in addition to the diagnosis of leukemia, this CNN can be trained to recognize more types of bone marrow metastasis for solid tumors.

Despite the encouraging performance of the deep learning model, this study has several limitations. (1) The presented model needs to be further validated by prospective studies. (2) The bone marrow images were collected from a single medical center, and the examples of some types of cells are limited; therefore, multi-center studies are needed to further develop the diagnosis system to diagnose more types of leukemia, especially AML. (3) The diagnostic performance of the proposed automated diagnosis system needs to be evaluated in clinical work.

## Conclusion

Our findings suggest that artificial intelligence algorithms may successfully assist hematologists in morphological diagnosis of leukemia in real clinical scenarios. In the future, we will collect more cells and establish a larger leukemia database to train the CNN and test its performance in leukemia diagnosis by comparing it with the performance of hematologists who are experts at morphological diagnosis.

## Data Availability Statement

The raw data supporting the conclusions of this article will be made available by the authors, without undue reservation.

## Ethics Statement

The studies involving human participants were reviewed and approved by The institutional review board of the Shanghai Children's Medical Center. Written informed consent for participation was not provided by the participants' legal guardians/next of kin because: The samples in this research were samples in clinical routine examination and all children's information is confidential.

## Author Contributions

MZ designed the research. KW wrote the paper. MX, QS, BL, and LS designed the CNN and provided statistical analysis. LY and JYa annotated the cells. BD, HW, and JYu provided trial coordination. LZ and SS lead the research. All authors contributed to the article and approved the submitted version.

## Conflict of Interest

The authors declare that the research was conducted in the absence of any commercial or financial relationships that could be construed as a potential conflict of interest.
