# Supplementary material for: Development and Evaluation of a Leukemia Diagnosis System Using Deep Learning in Real Clinical Scenarios
Source: Front Pediatr. 2021 Jun 24;9:693676. doi: 10.3389/fped.2021.693676 (PMC8264256; doi:10.3389/fped.2021.693676)
Supplement: Supplementary file 1 [file Image_1.pdf]

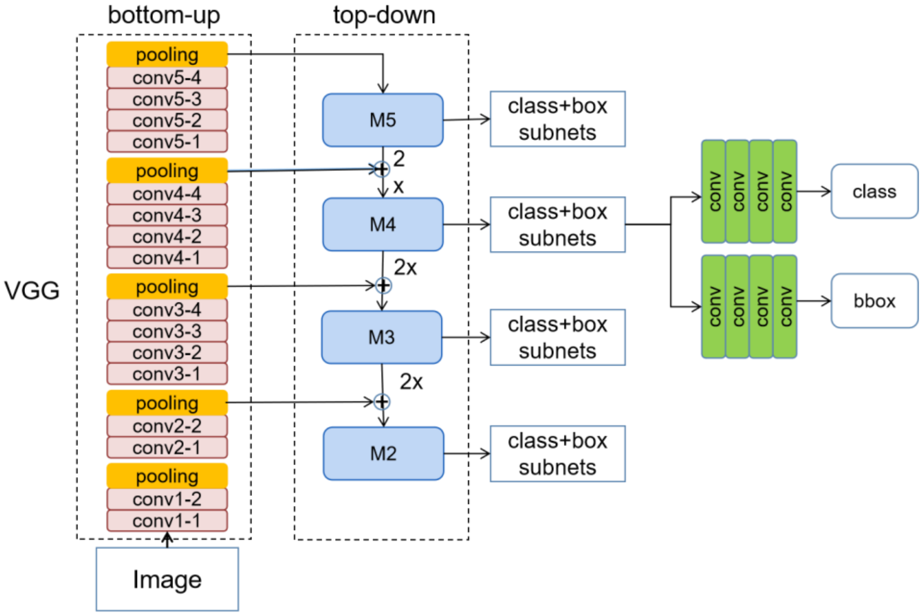

Figure S1. The framework of RetinaNet

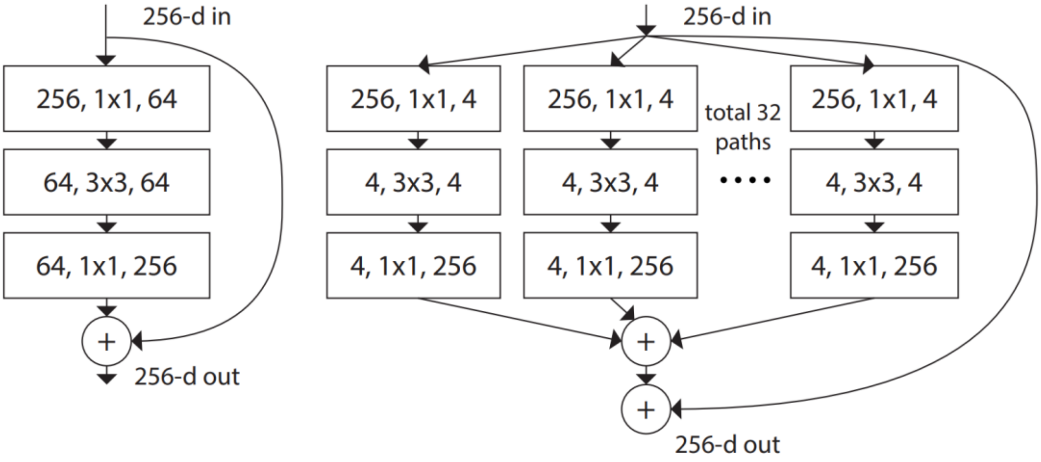

Figure S2. Left: ResNet block. Right: ResNeXt block

stage	output	ResNet-50	ResNeXt-50 (32×4d)
conv1	112×112	7×7, 64, stride 2	7×7, 64, stride 2
conv2	56×56	3×3 max pool, stride 2	3×3 max pool, stride 2
		[1×1, 64; 3×3, 64; 1×1, 256] ×3	[1×1, 128; 3×3, 128, *C*=32; 1×1, 256] ×3
conv3	28×28	[1×1, 128; 3×3, 128; 1×1, 512] ×4	[1×1, 256; 3×3, 256, *C*=32; 1×1, 512] ×4
conv4	14×14	[1×1, 256; 3×3, 256; 1×1, 1024] ×6	[1×1, 512; 3×3, 512, *C*=32; 1×1, 1024] ×6
conv5	7×7	[1×1, 512; 3×3, 512; 1×1, 2048] ×3	[1×1, 1024; 3×3, 1024, *C*=32; 1×1, 2048] ×3
	1×1	global average pool<br>1000-d fc, softmax	global average pool<br>1000-d fc, softmax

Figure S3. ResNet 50 and ResNeXt50(32x4d)
